# Immune cell therapy for hepatocellular carcinoma

**DOI:** 10.1186/s13045-019-0742-5

**Published:** 2019-05-29

**Authors:** Eishiro Mizukoshi, Shuichi Kaneko

**Affiliations:** Department of Gastroenterology, Graduate School of Medicine, Kanazawa University, Kanazawa City, Ishikawa 920-8641 Japan

**Keywords:** T cell, Dendritic cell, Chimeric antigen receptor, Cytokine-induced killer cell, Natural killer cell, Immunotherapy

## Abstract

Given the success of immune checkpoint inhibitors and chimeric antigen receptor (CAR) T cells in clinical settings, the host immune system plays an important role in the recognition and targeting of tumor cells in cancer immunotherapy. As a result, there have been numerous advancements in immune cell therapy using human immune cells. However, recent evidence suggests that one type of immunotherapy alone is not effective for the treatment of cancer, particularly solid tumors. Thus, effective immunotherapy combinations, such as the combination of checkpoint inhibitors and immune cell therapy, are needed. This review focuses on hepatocellular carcinoma among other solid tumors and discusses the current status and future of immune cell therapy in cancer immunotherapy.

## Background

Immunotherapy is considered the fourth pillar of cancer treatment after surgery, chemotherapy, and radiation therapy. Immune cells, antibodies, and checkpoint inhibitors are used in immunotherapy. Unlike conventional methods that target cancer cells, immune cell therapy, such as chimeric antigen receptor (CAR) T cell therapy and checkpoint inhibitors, is novel in that it makes use of the host immune system to treat cancer. As such, immune cell therapy may bring about a paradigm shift in the treatment of cancer.

Among the different immunotherapy strategies, we focused on hepatocellular carcinoma (HCC) to introduce the concept of immune cell therapy.

## Characteristics of HCC and immunotherapy

HCC is a malignant epithelial tumor arising from hepatocytes and is often associated with chronic hepatitis and cirrhosis caused by hepatitis B (HBV) or hepatitis C virus (HCV) infections [1]. Recent reports from developed countries suggested that metabolic disorders, such as diabetes, obesity, and fatty liver disease, are risk factors for HCC, indicating that it will become a public health concern [2].

The incidence of HCC is high in countries where the risk of HBV and HCV infections is also high. These countries include Japan; Korea; China; Taiwan; countries in Southeast Asia; part of Europe, including Italy and Spain; and countries in sub-Saharan Africa. Collectively, over 700,000 individuals die from HCC annually worldwide [3].

Treatments for HCC include hepatectomy, liver transplant, radiofrequency ablation (RFA), hepatic transarterial chemoembolization (TACE), chemotherapy, and molecular targeted therapy. Clinically, patients often undergo combinations of these treatments; however, these treatments are not effective for advanced forms of HCC [4]. In addition, even if the treatment is successful in eliminating HCC, the risk of recurrence is high because patients often have liver diseases that will eventually lead to the development of liver cancer. Therefore, a novel treatment strategy with different mechanisms from those of conventional treatments is needed to improve the prognosis of HCC. Immunotherapy is one such therapy that functions differently from conventional treatments. Recently, checkpoint inhibitors have been used successfully in cancer treatment; however, they are only effective in 10–40% of cases, and some cancers are resistant to checkpoint inhibitors [5, 6]. Indeed, previous studies found that checkpoint inhibitors do not elicit cancer-specific T cell responses in some patients and that cancer-specific T cells do not reach tumors in some cases [7]. In order to improve the effectiveness of checkpoint inhibitors, immune cell therapy may be an effective approach to induce cancer-specific T cells in patients who are resistant to checkpoint inhibitors. In addition to the need for novel treatments, HCC is a suitable model to study treatment effects on recurrence and long-term prognosis as HCC is associated with multiple recurrences and eventually leads to death.

## Targets for immune cell therapy in HCC

T cells are the main component involved in the antitumor immune response. The first step required for the development of T cell-based immune cell therapy is to identify antigens expressed on target tumors. Although HCC is not generally considered an immunogenic tumor, HCC patients who have a high level of lymphocyte infiltration in the tumors have a lower risk of recurrence and a better prognosis [8]. Furthermore, one study found that after RFA, patients with a high ratio of circulating tumor antigen-specific cytotoxic T lymphocytes (CTLs) in the peripheral blood have a significantly lower risk of recurrence than those with a low ratio of CTLs [9]. These findings suggest that HCC patients develop antitumor immunity that suppresses the progression of the disease. Studies in the past 10–15 years identified tumor-associated antigens (TAAs) in HCC and their respective T cell epitopes, thus confirming the presence of T cell-mediated immune response to HCC [10–21]. This also suggests that a novel immunotherapy for HCC can be established by developing a method to elicit potent antitumor responses.

Among the TAAs, the immune response to α-fetoprotein (AFP) has been studied in depth since CTL epitopes for AFP were identified at an early stage [10, 22]. AFP is a carcinoembryonic antigen and is produced in the body during fetal development. Although AFP is no longer produced soon after birth, it is produced again in HCC patients. Previous studies demonstrated that HCC patients are more likely to have T cells specific to AFP epitopes in the peripheral blood than healthy individuals and that the ratio of these T cells in the peripheral blood increases with cancer progression and after RFA and TACE [9, 23].

In addition to AFP, several TAAs have been identified for HCC. They include human telomerase reverse transcriptase (hTERT), melanoma antigen gene-A (MAGE-A), glypican-3 (GPC3), NY-ESO-1, cyclophyrin-B (Cyp-B), squamous cell carcinoma antigen recognized by T cells (SART), p53, and multidrug resistance-associated protein 3 (MRP3) [11, 12, 15, 16, 20, 21, 24, 25] (Table 1).

**Table 1:** Cytotoxic T cell epitopes expressed in hepatocellular carcinoma and their T cell receptors

Antigen	Frequency of occurrence (%)	Cytotoxic T cell epitope	HLA restriction	Year of report	Reporter [reference]	T cell receptor*	Year of report	Reporter [reference]
AFP	< 80	AFP_137_, AFP_158_	A2	1999	Butterfield et al. [10]	AFP_158_	2018	Docta et al. [26]
		AFP_325_, AFP_542_, AFP_357_, AFP_403_	A24	2006	Mizukoshi et al. [14]	AFP_357_	2017	Nakagawa et al. [27]
NY-ESO-1	< 50	NY-ESO-1_157_	A2	2004	Korangy et al. [11]	NY-ESO-1_157_	2014	Robbins et al. [28]
MAGE-A	< 80	MAGE-1_161_, MAGE-3_271_	A1, A2	2004	Zerbini et al. [12]			
		MAGE-10_254_	A2	2005	Bricard et al. [13]			
SSX-2	< 50	SSX-2_41_	A2	2005	Bricard et al. [13]			
hTERT	< 80	hTERT_167_, hTERT_324_, hTERT_461_, hTERT_637_, hTERT_845_	A24	2006	Mizukoshi et al. [15]	hTERT_461_	2015	Mizukoshi et al. [29]
Glypican-3	< 70	GPC3_144_, GPC3_298_	A2, A24	2006	Komori et al. [16]	GPC3_367_	2015	Dargel et al. [30]
p53	100	p53_149_, p53_264_	A2	2006	Cicinnati, et al. [25]			
HCA661	Unknown	HCA661_110_, HCA661_246_	A2	2007	Pang et al. [17]			
MRP3	< 55	MRP3_503_, MRP3_692_, MRP3_765_	A24	2008	Mizukoshi et al. [18]			
HCA587	< 70	HCA587_140_, HCA587_144_, HCA587_248_	A2	2008	Xing et al. [19]			
SART2	100	SART2_93_, SART2_161_, SART2_899_	A24	2012	Mizukoshi et al. [20]			
SART3	100	SART3_109_, SART3_315_	A24	2017	Kaji et al. [21]			

*AFP* alpha-fetoprotein, *MAGE* melanoma-associated antigen, *SSX-2* synovial sarcoma/X breakpoint-2, *hTERT* human telomerase reverse transcriptase, *HCA* hepatocellular carcinoma-associated antigen, *SART* squamous cell carcinoma antigen recognized by T cell

*The epitopes recognized by cytotoxic T cell receptor were described

## Antitumor immune response in HCC patients

Identification of CTL epitopes has led to the development of cancer immunotherapy. Furthermore, it is essential to understanding the mechanisms underlying immune response in HCC patients. One study examined the response of CTLs from HCC patients to several TAA-derived epitopes using enzyme-linked immunospot (ELISPOT) assay. The ratio of TAA-specific CTLs in peripheral mononuclear cells (PBMCs) of HCC patients ranged from 10 to 60.5 cells/300,000 PMBCs, and only 3–19% of patients had CTLs specific to the epitopes [31]. Immune responses in these ranges are lower than those against virus-derived foreign antigens.

Furthermore, another study examined CTL response using ELISPOT and tetramer assays and identified the presence of non-functional CTLs that bind to antigen epitopes but do not produce cytokines [18]. This demonstrated that as with other types of cancers, host immune response alone is insufficient to eliminate HCC. Thus, there is a need for additional interventions such as immune cell therapy. The following section describes the types of immune cell therapy that have been investigated for the treatment of HCC.

## Activated lymphocyte therapy

Several forms of immune cell therapy have been evaluated for the treatment of cancers. They include immunomodulators, such as OK432; cytokine therapy using interferons (IFN) and interleukins (IL); and lymphokine-activated killer (LAK) and cytokine-induced killer (CIK) cell therapies. Haruta et al. examined two adaptive cell transfer (ACT) techniques for HCC, namely LAK cell therapy and tumor-specific CTL therapy, and demonstrated CTL therapy to be effective as 3 of 18 patients achieved complete response (CR) and 2 of 18 patients achieved partial response (PR) [32]. Moreover, Takayama et al. used LAK cells as an adjuvant to surgery and reported that patients who were administered activated lymphocytes had a 5-year recurrence-free survival rate of 38% compared with 22% for those who did not receive the treatment [33].

CIK cell therapy has also been examined in numerous studies as immune cell therapy for HCC based on adaptive cell transfer [34–37]. CIK cells are isolated from PMBCs of patients, grown ex vivo, and cultured with a cytokine cocktail that produces cells with highly potent antitumor activity [36, 38]. Lee et al. found that CIK cell therapy improved the overall survival (OS) of patients when used in combination with either RFA or TACE [36, 37]. In addition, a phase II non-randomized study demonstrated that the addition of CIK cell therapy to a standard therapy improved OS and progression-free survival (PFS) [35]. These studies suggest that immune cell therapy is effective in reducing the recurrence rate, which is typically high for HCC patients following curative treatment.

## Natural killer cell therapy

Natural killer (NK) cells play an important role in the innate host immune response against viruses and tumors. The frequency and function of NK cells in the peripheral blood and liver are associated with recurrence and survival rates of patients with resectable HCC [39–41]. Thus, hepatic NK cells are thought to play an important role in mediating the immune function of the liver and immunological defense mechanisms against HCC [42].

Several clinical studies have demonstrated the efficacy of allogenic NK cells in adoptive immunotherapy for solid tumors, including HCC [43–46]. In particular, the combination of percutaneous cryoablation and NK cell therapy was found to be effective in prolonging the PFS of patients with advanced HCC [43]. Furthermore, multiple administration of allogenic NK cells was reported to improve the prognosis of advanced forms of HCC [43] and pancreatic cancer [44].

In addition to these studies, several approaches using genetic modification techniques have been developed to improve the specificity and efficacy of NK cell cytotoxicity to tumor cells. For example, the approach using CAR for T cells (described in a later section) has also been applied to NK cells, improving the specificity and efficacy of NK cell therapy [47–49]. CAR-NK cells reportedly reduce the risks of autoimmune response and neoplastic transformation because they have a shorter lifetime than CAR-T cells. In addition, cytokines released from NK cells, such as IFN-γ and granulocyte-macrophage colony-stimulating factor (GM-CSF), are considered safer than the cytokine storm that results from CAR-T cell therapy [50].

Among genetically modified NK cells, GPC3-specific CAR-NK-92 cells were reported to have high antitumor activity against HCC xenografts expressing both low and high levels of GPC3. The specificity of GPC3 CAR-NK-92 cells was confirmed by demonstrating that they are not cytotoxic to GPC3-negative HCC [51]. Clinical trials are currently underway to examine the safety and efficacy of CAR-NK cells [52, 53]. If successful, NK cell therapy may be used clinically for the treatment of solid tumors.

## Dendritic cell therapy

Dendritic cells (DCs) are the most potent antigen-presenting cells in the body. Upon recognition of antigens, DCs are activated and mature to enhance the antitumor immune response via T cells and NK cells [54, 55]. However, host immune systems involving DCs are restricted in tumors due to several mechanisms, including the low number of DCs in the tumor, reduced ability for antigen presentation, and limited access to tumor antigens [54]. Recent advancements in cell culture techniques revealed that GM-CSF and IL-4 trigger monocytes in the peripheral blood to induce significant activation of DCs. Therefore, artificially induced DCs can be administered intratumorally or subcutaneously to effectively stimulate DC-mediated host immune responses. In addition, current studies are examining the use of toll-like receptor (TLR) agonists, TAAs, and TAA-derived peptides as antigens to induce mature DCs that have potent antigen-presenting activity.

In the USA, a DC vaccine called sipuleucel-T was approved by the Food and Drug Administration (FDA) for use in patients with metastatic prostate cancer. Sipuleucel-T is a cell product that was developed by culturing DCs with a tumor antigen (prostatic acid phosphatase (PAP) fusion protein), and has been reported to prolong survival by approximately 4 months in a phase III trial [56].

Numerous DC-based immunotherapy techniques have been examined for HCC [57–65]. Although the majority of these techniques stimulate mature DCs before administration using TAA-derived proteins, TAA-derived peptides, or tumor lysates, DCs can be administered intratumorally without additional stimulation by antigens [57]. Among them, methods using antigen-derived peptides and proteins have the limitation to induce broad immune responses, and therefore, the methods by fusing tumor lysates with dendritic cells have been developed as a tumor vaccine. These techniques may be able to induce antitumor immunity against unknown antigens and their T cell epitopes [66, 67]. Other techniques have also been examined, including the re-administration of TAA-specific T cells into the body upon stimulation with ex vivo*-*induced DCs [65], and re-administration of DCs and CIK cells into the body after co-stimulation [58, 60]. Clinical trials and meta-analyses suggested that these DC-based strategies are effective in prolonging PFS and OS [68].

## Tumor-infiltrating lymphocyte therapy

Tumor-infiltrating lymphocyte (TIL) therapy is based on the administration of tumor-specific T cells, which have been isolated and cultured ex vivo from lymphocytes that have infiltrated resected tumors. Rosenberg et al. infused TILs into patients with advanced malignant melanoma, and 49–72% of patients achieved either CR or PR [69]. Furthermore, they demonstrated that patients who achieved CR were more likely to survive longer, suggesting that TIL therapy is effective for malignant melanoma. The antitumor effects of TILs may be attributed to TILs containing polyclonal T cells, both CD4^+^ and CD8^+^ T cells, and T cells that are specific to neoantigens. Overall, the development of TIL therapy confirmed that immunotherapy using cancer-specific T cells is clinically effective. Although the efficacy of TIL therapy was demonstrated in clinical trials for malignant melanoma, it has not been applied to the treatment of other types of cancers, including HCC, as it is challenging to culture cancer-specific TILs. Gene-modified T cell therapy can overcome these limitations associated with TIL therapy.

## Gene-modified T cell therapy

**Fig. 1:**
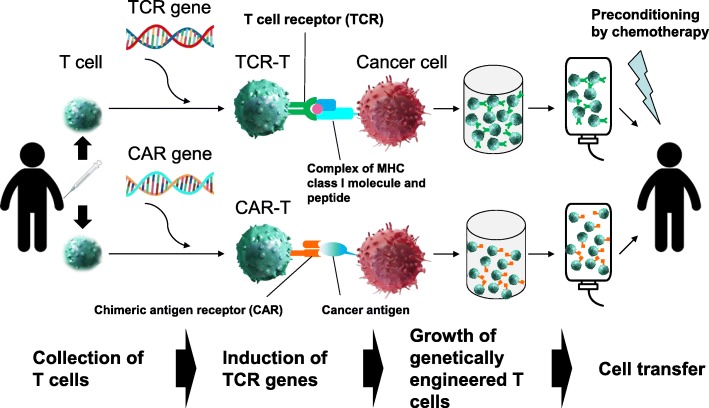
Overall picture of cancer immunotherapy using gene-modified T cells. This figure shows two methods of developing gene-modified T cells: one is based on the use of tumor antigen-specific TCRs from tumor-specific T cells or their clones which recognize the complex of MHC class I molecule and TAA-derived peptide, and the other is based on the use of CAR

Gene-modified T cell therapy has been developed as a method to deliver T cells that are specific to different types of cancers. It uses T cells that are genetically engineered to produce T cell receptors (TCRs) that recognize tumor antigens and their epitopes [70, 71]. Currently, there are two methods of developing gene-modified T cells: one is based on the use of tumor antigen-specific TCRs from tumor-specific T cells or their clones, and the other is based on the use of CAR (Fig. 1). The extracellular portion of CAR is a single-chain antigen recognition receptor composed of the variable regions of heavy and light chains of a monoclonal antibody specific to the tumor surface antigen, and the intracellular portion of CAR is created by binding of co-stimulatory molecules to the intracellular portion of TCR.

## CAR-T cell therapy

CAR-T cells are T cells modified by viral vectors to express CAR [72–74]. CAR-T cells are not limited by human leukocyte antigen (HLA) because the antigen recognitions site of CAR-T cells consists of monoclonal antibodies that specifically recognize tumor surface antigens.

CD19-CAR-T cell therapy was reported to be effective in a clinical trial as a treatment for recurrent and refractory acute lymphocytic leukemia (ALL) [75]. Similarly, in a trial on 16 patients with refractory ALL, 88% of the patients achieved CR [76], and in a subsequent trial with 53 patients, 83% of patients achieved CR by CD19-CAR-T cell therapy [77]. Moreover, in a global, multi-center trial for recurrent and refractory ALL including 75 patients, 81% achieved remission [78]. CD19-CAR-T cell therapy also led to an approximately 50% CR rate in patients with recurrent and refractory B cell non-Hodgkin’s lymphoma [79, 80]. Thus, CD19-CAR-T cell therapy may be highly effective, and two products have been approved by the FDA and are used clinically.

## TCR-engineered T cell therapy

TCR-engineered T (TCR-T) cells are produced by modifying T cells with the gene of TCR to specifically recognize the complex of tumor surface antigen peptides and major histocompatibility complex (MHC) molecules. Thus, TCR-T therapy is only effective if tumor cells express target antigen epitopes and MHC molecules. On one hand, HLA restriction needs to be taken into account because there are a variety of human MHC molecules, but all tumor-derived proteins that are processed by proteasomes can be targeted because the antigen itself does not need to be expressed on the cell surface. Therefore, many antigens can be targeted by TCR-T cell therapy. Although there are no commercially available TCR-T cell products, many have been tested in clinical trials (Table 2) [70, 71, 81–90]. Most have been tested against malignant melanoma, but few have been tested against breast cancer, esophageal cancer, or synovial sarcoma. Based on the Response Evaluation Criteria in Solid Tumors (RECIST) criteria, patients undergoing TCR-T cell therapy achieved CR and PR.

**Table 2:** Clinical effects and adverse events of T cell receptor gene-modified T cell therapies

Antigen	Type of cancer	Type of T cell receptor	Effects	Adverse events	Year of report	Reporter [reference]
MART-1	Malignant melanoma	Wild type	1/15 PR	Fever, fatigue et al.	2006	Duval et al. [81]
MART-1	Malignant melanoma	Wild type	2/17 PR	Not reported	2006	Morgan et al. [70]
MART-1	Malignant melanoma	High affinity	66/20 PR	Disorders of the skin, eye, and inner ear	2009	Johnson et al. [71]
MART-1	Malignant melanoma	High-affinity modification	9/13 tumor regression	Respiratory failure	2014	Chodon et al. [82]
gp100	Malignant melanoma	Mouse origin	1/16 CR, 2/16 PR	Not reported	2010	Davis et al. [83]
p53	Breast cancer, esophageal cancer, malignant melanoma	Mouse origin	1/10 PR	Not reported	2010	Davis et al. [83]
CEA	Colon cancer	Mouse origin	1/3 PR	Colitis	2011	Parkhurst et al. [84]
NY-ESO-1	Malignant melanoma	High-affinity modification	2/11 CR, 3/11 PR,	GVHD, Skin erythema, diarrhea, decrease in blood pressure	2011	Robbins et al. [85]
	Synovial sarcoma		4/6 PR			
	Myeloma		14/20 CR, 4/20 PR		2015	Rapoport et al. [86]
MAGE-A3	Malignant melanoma, myeloma	High-affinity modification	Not evaluated	Cardiogenic shock (death)	2013	Cameron et al. [87]
MAGE-A4	Esophageal cancer	Wild type	3/10 long survival	Not reported	2015	Kageyamama et al. [88]
WT-1	MDS, AML	Wild type	2/8 transient blast loss	Not reported	2017	Tawara et al. [89]
Tyrosinase	Malignant melanoma	Wild type	1/3 PR	Vitiligo	2018	Moore et al. [90]

*MART* melanoma antigen recognized by T cells, *CEA* carcinoembryonic antigen, *MAGE* melanoma-associated antigen, *CR* complete response, *PR* partial response, *MDS* myelodysplastic syndrome, *AML* acute myelogenous leukemia, *GVHD* graft versus host disease

## Harvesting TCRs specific to HCC antigens

There are several methods to harvest antigen-specific TCRs. One method is to establish a clone of antigen-specific T cells from tumor-infiltrating lymphocytes or PBMCs of cancer patients and subsequently clone TCRs from these T cells. However, the process of developing T cell clones is time-consuming, and the end product is limited to TCRs that originate from T cells that can be cloned. In other words, even if a TCR has potent antitumor activity, it may not be produced using this method if there are few T cells that express this particular TCR or if T cells expressing the TCR have limited proliferative capacity to establish clones. In order to overcome this limitation, techniques have recently been developed to clone TCRs from a single T cell [91, 92]. These techniques enable rapid cloning of TCRs at a single-cell level, resulting in the production of TCRs that cannot be harvested using the conventional method. Using these techniques, cloning of TCRs that bind to HCC-target proteins, including AFP, hTERT, MAGE, and NY-ESO-1, is possible [27–30] (Table 1).

Most TCRs that are isolated from lymphocytes of cancer patients have a low affinity for antigens. Tumor antigens, including differentiation antigens, such as gp100 and MART-1 for malignant melanoma; cancer/testis antigens, such as MAGE3 and NY-ESO-1; and overexpressed antigens, such as carcinoembryonic antigen (CEA) for colon cancer, are self-antigens that are expressed by normal cells. Thus, T cells harboring TCRs with a low affinity for these antigens typically remain in the body by negative selection in the thymus. Recent studies suggest that gene-modified T cell therapy using these low-affinity TCRs has limited antitumor effects. Several techniques have been developed in order to overcome this limitation, including a technique to artificially modify TCRs to make them high-affinity receptors [26] and a technique to immunize HLA transgenic mice using target antigens to isolate mouse-derived high-affinity TCRs [93].

## Perspective of gene-modified T cell therapy for HCC

We will first discuss preclinical studies on gene-modified T cell therapy for HCC. These studies used antigens and epitopes from HCV and AFP [93, 94]; TCR genes for HCV antigens and epitopes were isolated from human T cells, whereas those for AFP antigens and epitopes were isolated from human and murine T cells. One study examined the effect of TCR-T cell therapy using TCRs that recognize HCV and AFP-derived epitopes. The study used a super immunodeficient mouse model to grow HepG2 cells expressing the target antigens and demonstrated that TCR-T cell therapy was effective against HepG2 tumors in vivo. Future clinical trials may reveal that the treatment is also clinically effective in humans. Indeed, a phase I clinical trial is underway to examine TCR-T cell therapy that targets AFP in patients with advanced HCC (ClinicalTrials.gov identifier: NCT03132792). Regarding CAR-T cell therapy, one study used GPC-3 as a target in HCC, which prolonged the survival of mice harboring GPC-3 positive hepatic tumors [95].

In addition to GPC-3, mucin 1 (MUC1) and epithelial cell adhesion molecule (EpCAM) are considered to be good targets for CAR-T cell therapy in HCC patients. CAR-T cells that target these antigens are currently being developed for other cancer types [96, 97]. These CAR-T cells are also being tested for HCC in clinical trials (ClinicalTrials.gov identifier: NCT02587689, NCT03013712, NCT02729493, etc.) [97, 98], and positive outcomes are expected. Discovery of novel HCC-specific surface antigens may lead to the development of CAR-T cell therapy based on antibodies that recognize such antigens.

## Side effects of gene-modified T cell therapy

Many studies to date have described side effects associated with gene-modified T cell therapy. In CAR-T cell therapy, cytokine release syndrome (CRS) involving IFN-γ and interleukin-6 (IL-6) occurs in ALL patients due to enhanced immune cell activation [99–101]. Neurotoxicity is also one of the characteristic and important side effects of CAR-T cell therapy. Recent studies are revealing pathophysiology and risk factors of CRS and neurotoxicity [102]. The onset of these side effects is thought to be deeply involved in the process of antigen recognition and proliferation of CAR-T cells, but in studies using xenogeneic mouse models, monocyte/macrophage also plays a role in the onset of these pathologies [103]. Steroid and the antibody against the IL-6 receptor (tocilizumab) were effective for the treatment of CRS [104, 105]. On the other hand, in addition to IL-6, the involvement of IL-1 is the onset of CRS or cause of severe neurotoxicity is being clarified, and treatment with IL-1 blockade using anakinra is expected [102, 103]. Tumor lysis syndrome was also reported as a consequence of rapid and marked lysis of tumor cells. Thus, reduction of tumor size is recommended prior to initiating CAR-T cell therapy [106].

A clinical trial of TCR-T cell therapy for melanoma-associated antigens demonstrated that damage to normal melanocytes leads to the development of dermatitis, uveitis, and hearing impairments. TCR-T cell therapy targeting CEA also targeted CEA on normal intestinal epithelial cells and led to severe colitis [107]. Furthermore, TCR-T cell therapy targeting MAGE-A3 led to life-threatening central nervous system disorders and cardiomyopathy [108, 109]. In the case of myopathy, there was notable damage to myocardial cells not expressing MAGE-A3, and the damage was a consequence of TCR recognizing epitopes from titin, which is a structural protein of striated muscle. Studies are currently ongoing to address these issues with side effects as there are currently no established methods to accurately predict these adverse events prior to treatment. In most cases, HCC patients have reduced liver function because they often have chronic hepatitis or cirrhosis. Future studies should also focus on strategies to prevent and treat associated side effects in order for gene-modified T cell therapy to be widely used in the treatment of HCC.

## Immunosuppression and its solution in tumor microenvironment

In the field of cancer immunotherapeutic research, immunosuppressive mechanisms by cancer cells are becoming clear. Regulatory T cells (Tregs), myeloid-derived suppressor cells (MDSCs), and tumor-associated macrophages (TAMs) are known as cells that suppress host antitumor immunity, and these cells are increased in HCC patients and involved in tumor progression [110–112]. The tumor microenvironment is immunosuppressed by such immunosuppressive cells and cytokines such as TGF-β, IL-10, IL-6, and VEGF, and the mechanism is diverse.

In the liver, it has been reported that sinusoidal endothelial cells induce immune tolerance against CD8-positive T cells against TAAs released from cancer cells that have undergone apoptosis [113]. In addition, sinusoidal endothelial cells have been reported to contribute to the immunosuppressive environment in the liver by inducing Tregs or PD-L1 through membrane-bound TGF-β [114]. Furthermore, liver stellate cells are present in the liver, and in HCC patients in which the cells are activated, an immunosuppressive environment for the tumor is induced and reported to have a poor prognosis [115]. Activated stellate cells have been reported to induce monocytes to an immunosuppressive phenotype, MDSCs, T cell dysfunction, and apoptosis via PD-L1 expression [116].

Recent findings have reported the methods for improving the immunosuppressive environment in such a tumor microenvironment. Lenvatinib has been reported to enhance the therapeutic effect of immune checkpoint inhibitors by reducing TAMs locally at the tumor and enhancing antitumor immunity via interferon (IFN) signal [117]. In fact, also in human clinical trials, the efficacy of the combination therapy of lenvatinib and pembrolizumab has been reported [118]. Besides, the efficacy of the combination of VEGF inhibitor (bevacizumab) and anti-PD-L1 antibody (atezolizumab) for HCC has been reported [118]. Because VEGF increases TAMs and Tregs and enhances the expression of immune checkpoint molecules including PD1 molecules of CD8-positive T cells [119, 120], combination therapy of VEGF inhibitors and anti-PD-1 antibodies makes sense. It is expected that multiplex immunotherapy combining such molecular targeted drugs with immunotherapy will be increasingly developed in the future.

## Conclusion

As discussed in this review, studies on antitumor immunity have advanced rapidly in recent years and many of the findings are currently being applied clinically. These advancements will likely have a significant impact on immunotherapy for solid tumors, and future developments of immune cell therapy, particularly gene-modified T cell therapy, such as CAR-T or TCR-T cell therapies, are highly anticipated for the prevention of recurrence and as novel treatment strategies for HCC. Future studies should focus on the identification of highly immunogenic TAAs and their respective T cell epitopes, establishment of safer and more effective gene modification techniques for T cells, and an improved understanding of the mechanisms underlying the suppression of antitumor effects by tumor cells. These studies will lead to the development of novel and multiplex immunotherapy strategies for the future of cancer treatment.

## Data Availability

The material supporting the conclusion of this review has been included within the article.

## Abbreviations

ACT: Adoptive cell transfer
AFP: Alpha-fetoprotein
CAR: Chimeric antigen receptor
CIK: Cytokine-induced killer
CTL: Cytotoxic T lymphocyte
Cyp-B: Cyclophyrin-B
ELISPOT: Enzyme-linked immunospot
GPC3: Glypican-3
HBV: Hepatitis B virus
HCC: Hepatocellular carcinoma
HCV: Hepatitis C virus
hTERT: Human telomerase reverse transcriptase
IFN: Interferon
LAK: Lymphokine-activated killer
MAGE: Melanoma antigen gene
MRP3: Multidrug resistance-associated protein 3
NK: Natural killer
SART: Squamous cell carcinoma antigen recognized by T cells
TAA: Tumor-associated antigen
TACE: Transarterial chemoembolization
TCR: T cell receptor
TIL: Tumor-infiltrating lymphocyte
